# CD28-costimulated CD19 CAR-T cells for pediatric mature non-Hodgkin B-cell lymphoma

**DOI:** 10.1038/s41409-025-02615-0

**Published:** 2025-04-23

**Authors:** Alon Abramovich, Etai Adam, Adi Shapira, Daphna Hutt, Orit Itzhaki, Bella Bielorai, Amos Toren, Elad Jacoby

**Affiliations:** 1 Division of Pediatric Hematology and Oncology, The Edmond and Lily Safra Children’s Hospital, Sheba Medical Center, Tel Hashomer, Israel; 2 Faculty of Medical & Health Sciences, Tel Aviv University, Tel Aviv, Israel; 3 Department of Pediatric Hematology-Oncology, Rambam Medical Center, and The Bruce Rappaport Faculty of Medicine, Technion-Israel Institute of Technology, Haifa, Israel; 4 Ella Institute of Immuno-Oncology, Sheba Medical Center, Tel Hashomer, Israel

**Keywords:** Phase II trials, Cancer immunotherapy

## Abstract

Children with relapsed or refractory (R/R) mature B-cell non-Hodgkin lymphoma (B-NHL) have a poor prognosis with approved therapies. Chimeric antigen receptor (CAR)-T cells are approved for adults with R/R B-NHL, but pediatric data is lacking. We report on 13 children with R/R mature B-NHL enrolled on a clinical trial for CD19 CAR-T cells harboring CD28 costimulation. Twelve patients were infused with CAR-T cells, and one had progressed and died prior to infusion. Toxicities included cytokine release syndrome in 8 patients and neurotoxicity in 6, including two patients with grade 4 neurotoxicity. All patients responded to CAR-T cells, including a complete response in 6, complete metabolic response in 2 and partial response in four. The median event-free survival was 15.2 months and median overall survival was not reached. Outcome differed by disease type, as most patients with primary mediastinal B-cell lymphoma had long term remissions, while only two of seven patients with Burkitt lymphoma were long term survivors. Thus, initial response may suffice for certain patients, but further consolidative strategies should be studied in patients with R/R Burkitt lymphoma.

## Introduction

Pediatric mature non-Hodgkin lymphoma (NHL) are mostly high-grade aggressive lymphomas of B-cell origin, including Burkitt lymphoma (BL), diffuse large B-cell lymphoma (DLBCL) and primary mediastinal B-cell lymphoma (PMBCL) [1–3]. These lymphomas frequently present with disseminated disease, often involving the bone marrow or central nervous system. The up-front therapy for children with these aggressive lymphomas is typically short-term, intensive, multiagent chemotherapy with the addition of rituximab [4, 5]. The 5-year outcomes are excellent for this group of patients with most histologies [6]. Children with relapsed or refractory (R/R) mature non-Hodgkin B-cell lymphoma are treated with intensive chemotherapy and autologous stem cell transplantation (ASCT), and usually have a poor prognosis [3, 7, 8]. The outcomes of relapsed disease in children after modern rituximab-based therapy is especially poor [9].

Since first being reported in 2010, CD19-directed chimeric antigen receptor (CAR) T cells have become standard-of-care in 2nd or 3rd line adult mature B-NHL. Adults with R/R DLBCL and PMBCL treated with either Axicabtagene ciloleucel, Lisocabtagene maraleucel and Tisagenlecleucel have a complete response rate of 40–60% and 60–70%, respectively, confirmed also by real-world studies [10–14]. Reports in BL are scarce, but response has been demonstrated in patients treated with CD19 CAR T cells [15–17].

Given the excellent outcomes in upfront protocols, the rarity of pediatric R/R B-NHL resulted in slow clinical development. Data in pediatric mature B-cell lymphoma is scarce and limited to a single study from China [18], and two early reports of clinical trials in abstract form [19, 20]. In general, pediatric development of several products has not matured into clinical approval [21, 22]. We hereby report an initial cohort of pediatric ( < 19 years old) patients with relapsed/refractory mature B-cell lymphomas treated on a prospective clinical trial.

## Methods

### CAR production and study oversight

We conducted a single-center phase 2 clinical trial for CD19-expressing hematologic malignancies, in a tertiary hospital serving national and international patients, between 2016 and 2024. Results of pediatric ALL and adult NHL have been previously reported [23, 24]. Briefly, autologous peripheral blood mononuclear cells were leukapheresed, activated and transduced with an FMC63-CD28-CD3zeta CAR encoded on an MSGV retrovirus (kindly provided by Dr. Steve Rosenberg). Patients received lymphodepletion with fludarabine (25 mg/m^2^/day for 3 days) and cyclophosphamide (900 mg/m^2^ for 1 day) prior to an infusion of cells, per protocol guidelines [25]. Release criteria for all products include viability ≥70%, CAR transduction ≥15%, and interferon-gamma release potency assay, and standard sterility and mycoplasma testing. A fresh CAR-T product was infused to all patients. In two cases, apheresis was cryopreserved and production was initiated after bridging chemotherapy. CAR-T cell expansion in the peripheral blood was monitored by flow cytometry (CD19 CAR detection reagent, Miltenyi Biotech, DE) or by qPCR previously described [25].

### Response and toxicity criteria

CAR-T related toxicity was graded per ASTCT guidelines [26]. All patients were evaluated for response using FDG-PET on day 28 post CAR-T infusion (± 5 days). Additional imaging were performed per physician’s request. For patients with bone marrow involvement, a bone marrow aspiration with flow-cytometry minimal-residual disease (MRD) was performed at the same time.

Response was evaluated according to Lugano classification criteria [27], the type of response was divided into four subgroups: Complete response (CR) was considered complete anatomical and metabolic response by PET-FDG. For patients with bone marrow involvement of Burkitt leukemia, absence of malignant cells by flow-cytometry MRD monitoring was necessary for definition of CR. Complete metabolic response (CMR) was defined as absence of PET-avid lesions with a partial anatomical response [27]. A partial response (PR) was defined as partial anatomic and metabolic response. Non-response (NR) was considered stable or progressive disease.

### Supportive care

All patients received prophylaxis for *P. jiroveci*. Anti-fungal and anti-viral prophylaxis was not mandatory but per physician’s discretion. Per our institutional guidelines, tocilizumab is given to any patient with grade 3 or greater cytokine release syndrome (CRS), and corticosteroids administered to patients with any kind of immune-effector cell associated neurotoxicity syndrome (ICANS) following CD28-costimulated CAR T-cells. Immunoglobulins were monitored at least monthly after CAR-T cell infusion, till recovery (measurable IgM levels). IVIg supplementation was given if IgG levels were lower than 400 mg/dL.

### Statistical analysis

Statistical analyses were performed using Prism v10.4 (GraphPad software). For event-free survival (EFS), an event was considered relapse or death of any cause. Overall survival (OS) and EFS were censored at last assessment date for patients without events or lost to follow-up.

## Results

### Patients characteristics

We describe 13 children with R/R non-Hodgkin lymphoma enrolled in a trial for the treatment of R/R lymphomas, 12 of whom were treated with CD19 CAR T cells in our institution. One patient did not start lymphodepletion due to disease progression. Patients included 7 patients with Burkitt lymphoma / leukemia, 4 patients with PMBCL and one patient with DLBCL and one had a composite lymphoma of PMBCL and DLBCL.

Patients characteristics are described in Table 1. The median age at CAR-T cell therapy was 11 years (range, 3.7–18.8). Patients had failed a median of 2 previous therapy lines. Responses to the prior therapy line before CAR-T cells included no response in 3 patients, partial response in 5, and complete response in 5. Of the latter, 3 had relapsed prior to CAR-T therapy and 2 were treated with no evidence of active disease. Overall 9 patients entered lymphodepletion with active disease, 1 patient had evidence of minimal residual disease in the bone marrow, and 2 patients were treated in remission. Five patients had elevated LDH prior to lymphodepletion, and two patients had low platelet counts (Table 1).

**Table 1:** Patients characteristics.

	Gender	Diagnosis	Age at Initial Diagnosis	Disease Site involvement		Prior therapy lines	Prior HSCT	Age at enrollment	Therapy before CAR T	Response to therapy before CAR T	Disease status pre LD	Disease sites prior to CAR T	Pre-LD LDH (U/L)	Pre LD Platelet (K/µL)	Pre LD Neutrophil (per µL)
				BM	CNS										
1	M	Burkitt	3.4	+	+	3		3.7	Blina.	PR	MRD	BM	220	122	1650
2	M	Burkitt	7.9			2		8.9	ICE	CR->relapse	*	*			
3	M	Burkitt	12.2	+		**1**		13.2	BFM90	CR->relapse	Active	Pancreas, skin, BM	830	191	1790
4	M	Burkitt	17.8			3		18.8	R-VICI	NR	Active	Peritoneum	929	221	7790
5	F	Burkitt	3.2	+	+	2		6.4	R-ICE	CR	NED	–	281	216	3210
6	F	Burkitt	7.4		+	2		8.3	R-ICE	CR	NED	–	251	235	2750
7	M	Burkitt	5.5	+		2		6.2	R-ICE	PR	Active	BM	3695	28	1320
8	M	DLBCL	5.6			3	+	7.4	Auto-HSCT	CR->relapse	Active	Mediastinum, Lungs	214	160	610
9	F	PMBCL	14.1			3		15.0	BV+Nivo	NR	Active	Mediastinum	394	349	3110
10	F	PMBCL	16.7			2		17.5	BV+Nivo	NR	Active	Mediastinum, Kidneys	301	269	6230
11	M	PMBCL	11.5	+		3		17.5	BV+Nivo	PR	Active	Mediastinum, BM, Kidneys, Pancreas, skull	261	231	2160
12	M	PMBCL	16.7			2		17.6	BV+Nivo	PR	Active	Mediastinum, Lungs	246	266	3070
13	M	DLBCL/ PMBCL	11.2			2		12.4	R-ICE	PR	Active	Mediastinum	427	307	5050

*DLBCL* diffuse large B-cell lymphoma, *PMBCL* primary mediastinal B-cell lymphoma, *BM* bone marrow, *CNS* central nervous system, *HSCT* hematopoietic stem cell transplantation, *LD* lymphodepletion, *LDH* lactate dehydrogenase, *Blina* blinatumomab, *R-ICE* rituximab, ifosphamide, carboplatin, etoposide, *R-VICI* rituximab, vincristine, idarubicin, ifosfamide, carboplatin, and dexamethasone, *BV+Nivo* brentuximab vedotin + nivolumab, *CR* complete response, *PR* partial response, *NR* non response, *MRD* minimal residual disease, *NED* no evidence of disease.

* Pt.2 passed away prior to lymphodepletion while CAR-T cell were being manufactured.

### CAR T therapy and toxicity

The median time between apheresis and CAR-T administration was 11 days (range 10–94). CAR T-cells were successfully manufactured in all patients but 1. That one patient, with relapsed Burkitt lymphoma, had progressive disease following apheresis and died of his disease prior to lymphodepletion, at which point CAR T cell production was stopped. The remaining twelve patients were each infused with 1×10^6^ CAR-T cells/kg and are evaluable for response. Eight patients developed CRS: one had grade 3, one had grade 2 and six had grade 1. Overall, the rate of CRS was 67%, and of grade 3 + CRS 8.3%. Six patients developed ICANS: four patients with grade 1 and two with grade 4. One patient (#4) with massive peritoneal involvement became unarousable on day +6 and developed generalized seizures. CT scan did not show brain edema. He was treated with high-dose steroids, levetiracetam and anakinra. Despite therapy the patient had prolonged clinical and EEG-evident encephalopathy and flaccid paralysis with a diffusely abnormal MRI of the brain 3 and 6 weeks after CAR T cells, and did not recover his baseline neurologic function. A second patient (#5) developed grade 4 ICANS on day +6 including confusion and seizures. Brain CT scan did not reveal any edema. She was treated with high-dose steroids and anti-epileptic drugs along with mannitol and hypertonic saline. Toxicity completely resolved on day +11. The rate of ICANS was 50%, with 16.6% grade 3 or above. Out of 3 patients with a history of CNS involvement, only 1 had grade 3 + ICANS. Overall one patient received tocilizumab, 5 received corticosteroids and 1 received anakinra for CAR related toxicity. Hematologic toxicity was observed in 11 patients: neutropenia was recorded at grade 2 (*n* = 1), grade 3 (*n* = 6) and grade 4 (*n* = 4). Two patients developed grade 4 thrombocytopenia. At day 28 25% of the patients had grade 3 or higher neutropenia, and 8.3% had grade 3 or higher thrombocytopenia. The only patient with prolonged neutropenia was referred to an allogeneic HSCT. B-cell aplasia was documented in all patients at day 28, and was ongoing for 2–6 months (Table 2). Patients were supplemented with IV immunoglobulins for 1–12 months. Two patients who had no further therapy after CAR-T cells experienced late infections, including COVID19 in one, and acute otitis media and herpes simplex in another.

**Table 2:** Patient-specific toxicity and outcome.

	Diagnosis	CRS grade	ICANS grade	ICHAT grade	Response 28 d	Further therapy	Duration of BCA (months)	Duration of IVIG supplementation (months)	Infections	Outcome
1	Burkitt	0	0	3	CR	Allo-HSCT in CR	1^a^	1^a^		Relapse, DOD
3	Burkitt	2	1	3	PR	—	N/a	2		Relapse, DOD
4	Burkitt	3	4	4	PR	—	2	2		Death in remission
5	Burkitt	1	4	0	CR	—	4	2	HSV, AOM	Prolonged CR
6	Burkitt	1	0	3	CR	—	6	12	COVID19	Prolonged CR
7	Burkitt	0	0	4	CR	Allo-HSCT in CR	1^a^	1^a^		Relapse, DOD
8	DLBCL	1	0	3	CR	Ibrutinib + Allo-HSCT for relapse	N/a	3		Alive after further therapy
9	PMBCL	1	1	4	PR	Nivolumab + Brentuximab	1	N/a		Alive after further therapy
10	PMBCL	0	0	2	CR	—	5	5		Prolonged CR
11	PMBCL	1	1	4	CMR	—	3	6		Prolonged CR
12	PMBCL	1	0	3	PR	—	2	2		Prolonged CR
13	DLBCL/ PMBCL	0	1	3	CMR	—	6	6		Prolonged CR

*CRS* cytokine release syndrome, *ICANS* immune-effector cell associated neurotoxicity syndrome, *ICHAT* Immune effector cell–associated hematotoxicity, *IVIG* intravenous immunoglobulins, *DLBCL* diffuse large B-cell lymphoma, *PMBCL* primary mediastinal B-cell lymphoma, *CR* complete response, *CMR* complete metabolic response, *PR* partial response, *NR* non response, *MRD* minimal residual disease, *NED* no evidence of disease, *HSCT* hematopoietic stem cell transplantation, *DOD* died of disease, *AOM* acute otitis media, *HSV* herpes simplex virus.

^a^data is limited due to fast referral to HSCT.

### Response

**Fig. 1:**
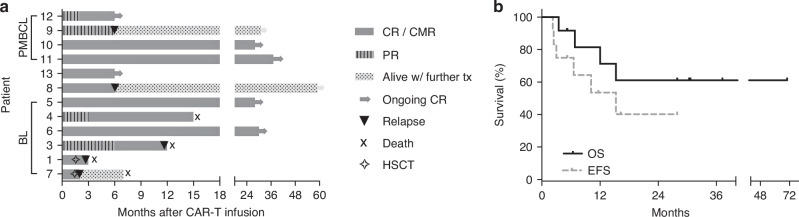
Outcomes of pediatric patients with mature non-Hodgkin lymphoma treated with CD19 CAR T cells. **a** swimmers plot depicting response, further therapy, relapse and mortality. **b** Kaplan-Meier curve of overall survival (OS, black line) and event-free survival (EFS, dashed grey line).

Eleven patients had evidence of CAR-T cell expansion in the peripheral blood, and all patients had subsequent B-cell aplasia. All 12 evaluable patients had a response to CAR-T cell therapy within 30 days (Fig. 1a): Six patients achieved complete response (CR), two patients (both with PMBCL) had a CMR and four had a partial response. Both patients with complete metabolic response had a complete response by 3–6 months. Two patients with a partial response treated for PMBCL and BL had progressed 6 and 12 months after treatment, respectively. Two additional patients with a partial response proceeded to complete response at 3 and 6 months evaluation.

### Relapse and survival

Two patients had undergone allogeneic-SCT in remission for Burkitt leukemia/lymphoma, using a total-body irradiation based conditioning. Both patients relapsed within 30 days of HSCT. One patient was referred to palliative care and died of his disease, and the second had a CD19-negative relapse and was referred to antibody-based therapy (inotuzumab and glofitamab) but eventually passed away of his disease. The remaining 10 patients had no subsequent therapy while in remission. Three of these patients relapsed: One with Burkitt lymphoma, who died of his disease (Fig. 1a). One patient with PMBCL who had a PR had relapsed and was treated with brentuximab vedotin and nivolumab (BV+Nivo). One patient with DLBCL relapsed at 6 months after having a complete response. This patient was found to have a germline ZAP70 mutation that is related to lymphoma risk, and was further treated with salvage therapy and an allogeneic hematopoietic stem-cell transplant (allo-HSCT), and is still alive.

One patient with BL had prolonged disability after severe neurotoxicity and had died in remission 15 months after CAR-T cell infusion. At last follow-up 8 patients are alive and 6 are in continuous complete remission without additional therapy (Fig. 1a).

The median event-free survival is 15.2 months and the median overall survival was not reached (Fig. 1b). With a median follow-up of 21.5 months, the estimated 2-year EFS is 40% and OS is 61%.

## Discussion

Despite being commercially available for adult B-NHL and pediatric ALL, data on CAR-T therapy in pediatric R/R B-NHL is lacking. We present our experience with CD19 CAR-T cell therapy for children with R/R mature B-NHL. Standard therapy for such patients includes multi-agent chemotherapy and rituximab, and typically consolidated with an autologous stem cell transplant. Response to salvage chemotherapy for R/R mature B-NHL is reported to be 50–75% [28], and many patients do not reach transplant [8]. Published data on children with first relapse of BL shows that survival with this approach is 30% or even lower [8, 9, 28, 29], especially when rituximab is administered in 1st line [8, 9], as is the current standard-of-care. Thus, R/R BL is a challenge requiring novel therapeutic measures.

Development of CD19 CAR T cells in adult NHL had brought CAR-T cells to approval in 2nd relapse and later in 1st relapse [11]. Interestingly, while in pediatric ALL the need for persistence has shown superiority of 4-1BB costimulated CAR-T cells, this is not the case for mature B-NHL. Moreover, non-randomized studies of 2nd relapse, CD28-costimulated CAR-T cells have shown better outcomes compared to 4-1BB costimulated CAR-T cells in adults with B-NHL [30, 31]. Our hypothesis was that the need for durable CAR-T cells in these diseases is limited, and that CD28 costimulation may overcome the aggressive nature of these lymphomas.

The overall response rate in our cohort was 100%. Despite high response rates, only two patients treated for BL survived long-term. Two patients had undergone consolidative allogeneic HSCT and relapsed within 1 month of transplant. One patient relapsed 1 year after therapy, and a fourth one had died of subsequent complications of CAR-T cells.

There are a few reports on CAR-T cells in pediatric patients with B-NHL. A study from Seattle Children’s Hospital reported on 8 children with R/R B-NHL treated with CD19 CAR T cells. Five patients responded, including two patients with DLBCL having a CR. Nevertheless, responses were not durable [19]. Tisagenlecleucel, a 4-1BB costimulated CD19 CAR-T product approved for R/R pediatric ALL and adult NHL, was studied in pediatric B-NHL, and early results were presented at the 2022 European Hematology Association annual meeting. Overall, 33 children and young adults with B-NHL were infused with tisagenlecleucel. The ORR was 32% and 7% had a CR, leading to a 12-month EFS and OS of 23% and 47%, respectively. Seven of the eighteen patients treated for BL were alive, though only 2 are in remission without further therapy [20]. In a report of 23 patients with R/R BL from a single center in China treated with CD19 CAR-T cells with 4-1BB costimulation, 9 had ongoing CR after CD19 CAR-T cells. The remaining patients had received subsequent CD22 and CD20 CAR-T cells. Altogether they report an 18-month progression-free survival of 78% [18]. All the aforementioned trials used 4-1BB costimulation. In three case reports in adult BL treated with CD28-costimulated CAR-T cells (two with FMC63 ScFv and one with a humanized ScFv), all 3 patients had achieved a CR, and two had long term remissions [15, 17]. A study of six adult patients with BL treated with a 3^rd^ generation CAR, combining CD28 and 4-1BB for costimulation reported 1 CR and 2 PR that were not maintained [16]. Altogether, data is inconsistent between trials, and awaits further confirmatory multi-center studies.

The toxicity in our cohort was significant, however high grade CRS was reported in 1 patient (8%) and high grade ICANS in 16% of patients. These outcomes are similar to other published cohorts of CD28 costimulated CAR-T cells [10, 11, 31].

Outcomes of R/R PMBCL in children are better compared to BL, with a 3-year EFS of 58% [32]. Novel therapies such as BV+Nivo are used in R/R PMBCL, and excellent outcomes were reported in adults [33]. In these reports, many patients underwent a consolidative HSCT, or required prolonged therapy for more than 1 year. Outcomes in patients with PMBCL treated in our study were better than with BL: Three patients in were remission without subsequent therapy, and all patients surviving. Importantly, all four patients with PMBCL had poor responses BV+Nivo prior to enrolling on our trial, suggesting CAR-T cells as either salvage therapy or consolidation for this approach.

Our data supports further development of CAR-T cells for pediatric B-NHL. Since numbers are small we cannot further analyze risk factors associated with benefit from this therapy. Large prospective studies have been difficult to complete with rarity of patients. Outcomes in R/R PMBCL are excellent, similar to adult data in this disorder [13], and perhaps current approval of commercial CAR-T cells for this disorder may be extended to pediatric and adolescent population. Expanding access of current approved CAR-T products to adolescents should be considered in such rare disorders. Long-term outcomes in patients with R/R Burkitt lymphoma are still not satisfactory. Current approaches with CD19 CARs for heavily relapsed patients still yield few long-term responders. Additional therapy, either through bi-specific CAR-T cells (NCT06508931), sequential infusions of CAR-T cells directed against different antigens [18], or addition of bispecific antibodies targeting CD20 [34] currently studied in children, may be future strategies fit for further investigation for treating children with relapsed disease.

## Data Availability

Data will be provided upon request.
